# Balkan Endemic Nephropathy: An Autopsy Case Report

**DOI:** 10.7759/cureus.12415

**Published:** 2021-01-01

**Authors:** George S Stoyanov, Ina Kobakova, Lilyana Petkova, Deyan L Dzhenkov, Hristo Popov

**Affiliations:** 1 General and Clinical Pathology/Forensic Medicine and Deontology, Medical University of Varna, Varna, BGR

**Keywords:** balkan endemic nephropathy, pathology, histology, interstitial nephritis

## Abstract

Balkan endemic nephropathy (BEN) is a form of interstitial nephritis seen only in certain geographical areas in the Balkan peninsula. Herein we describe the gross and histological changes in a diseased 42-year-old male Caucasian patient with BEN. All the changes fit the classically described alterations, with copper hue discoloration of the skin of the torso and orange discoloration of the soles and palms. Grossly, the kidneys were atrophic, with the left one weighing 31 grams and the right one 32 grams. Their surface was predominantly smooth with areas of fine granulations and cystic transformations. Histology revealed hyalinization of the glomeruli, predominantly in the external part of the cortex, severe vascular changes, interstitial fibrosis, and scant inflammatory cell infiltrate. The renal pelvis and ureters revealed multiple urothelial papillomas and atypical urothelial hyperplasia. BEN is only one geographical variant of interstitial nephritis caused by exposure to aristolochic acid. Other forms of this condition include Chinese herb nephropathy/aristolochic acid nephropathy, as well as several similar endemic conditions with a yet unestablished link to aristolochic acid.

## Introduction

Balkan endemic nephropathy (BEN), which was first described by Tanchev in 1956, is a form of interstitial nephritis, with severe gross reduction of renal parenchyma, acquired copper hue of the skin, and increased prevalence of urothelial papilloma of the ureters and urothelial malignancies [1]. In this condition’s original description, patients were noted to be from small villages, had a familial history of renal disease, the aforementioned acquired skin discoloration, including the palms and soles, normochromic anemia, absence of acute onset, albuminuria, hypertension, edema, absence of compensatory proteinuria, azotemia with rapid progression to severe uremia, and a mortality rate of more than 80% within the first year of diagnosis [1,2]. The age of onset varied from 30 to 60 years [1]. Despite the endemic nature of the condition in only a small part of the Balkan peninsula (located in Southeastern Europe), multiple studies have reconfirmed the original findings, with autopsy studies defining the condition as the nephropathy of the “smallest kidneys”, as the kidneys of the patients always weighed less than 50 grams, with some even being less than 15 grams (normal kidney weight ranges from 120 to 160 grams) [2]. We present the gross and histological characteristics of a patient referred for autopsy with BEN.

## Case presentation

At the time of death, the patient was 41 years old. At the age of 34 years, he had presented with anemia, hypertension, and uremia. Clinical examination and laboratory tests revealed severely decreased renal function and the patient was referred to hemodialysis. Due to the non-specific findings, the diagnosis of the patient was defined as chronic nephritis with end-stage kidney disease.

In the following years, the patient suffered recurrent thrombotic events of the arterio-venous fistula (AVF) formed for hemodialysis, a progression of the hypertensive state, severe episodes of anemia, and respiratory infections. During a hospital stay for the removal of the thrombotic AVF, for which the previous procedure of hemodialysis could not be performed properly, the patient presented with worsening uremia, severe anemia (hemoglobin levels of 58 mg/ml), and expired.

**Figure 1:**
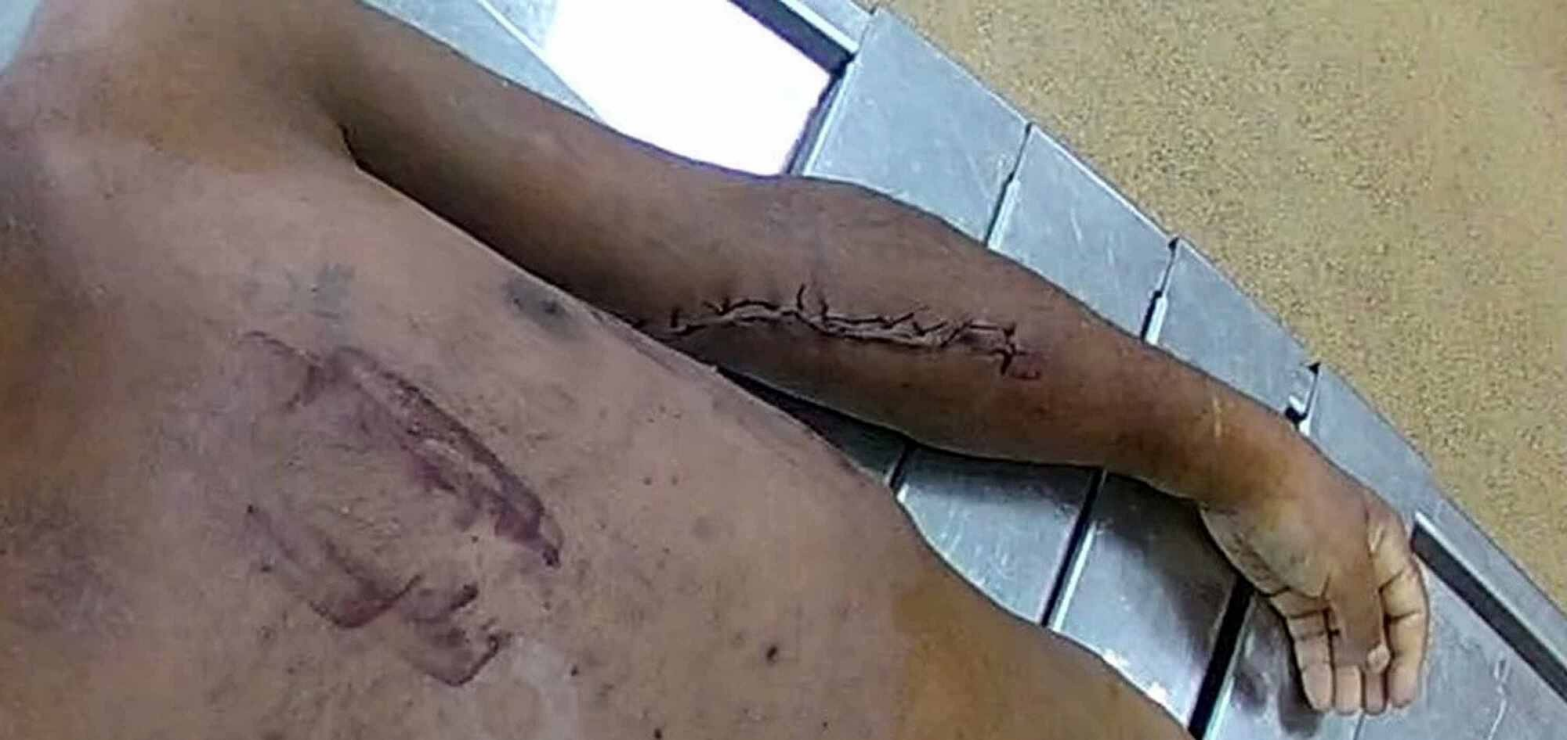
Copper hue discoloration of the skin, with orange discoloration of the palms The figure is a trimmed-out snapshot of the autopsy video recording. Note that the patient is Caucasian.

Due to the clinical diagnosis of chronic nephritis with end-stage kidney disease and the short hospital stay, the patient was referred for autopsy. Before the autopsy, a copper hue discoloration of skin and orange discoloration of the palms and soles was noted (Figure 1).

**Figure 2:**
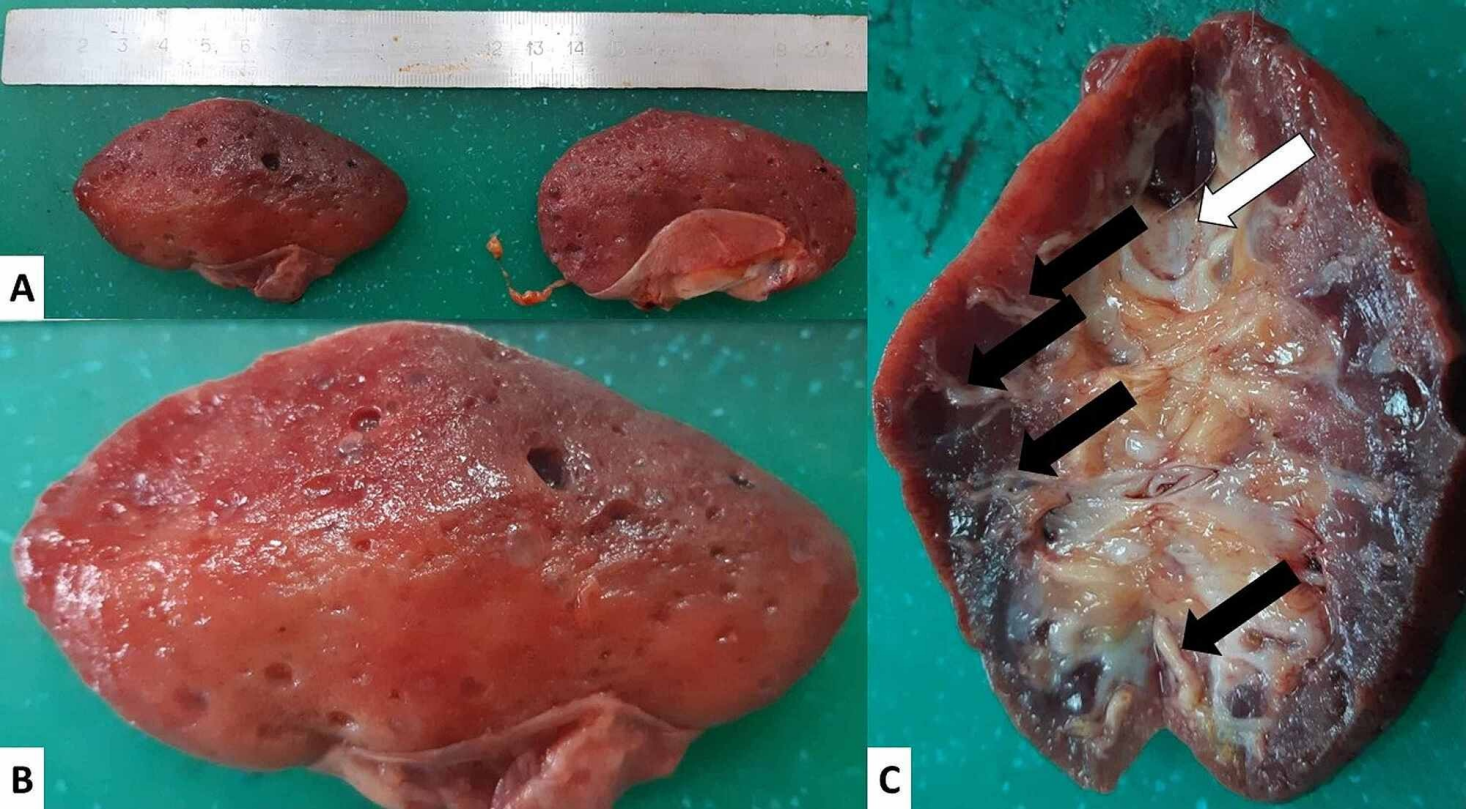
Gross kidney morphology (A) The two kidneys side by side; (B) the surface of the left kidney with cystic areas and finely granulated cortex and smooth cortical areas; (C) cross-section of the left kidney showing a thinned-out cortex, severe vascular changes (black arrows), and granulated area of the renal pelvis mucosa (white arrow)

The kidneys were of a grossly diminished size and weight (Figure 2). The left kidney weighed 31 grams and the right one weighed 32 grams. The renal surface was smooth, with areas of fine granulations and scattered superficial cysts. On cross-section, the renal parenchyma was severely thinned out, with prominent sclerotic and hyalinized blood vessels (Figure 2C). The renal pelvis and ureter mucosa had multiple areas of fine mucosal granulations and visible papillary growths (Figure 2C).

**Figure 3:**
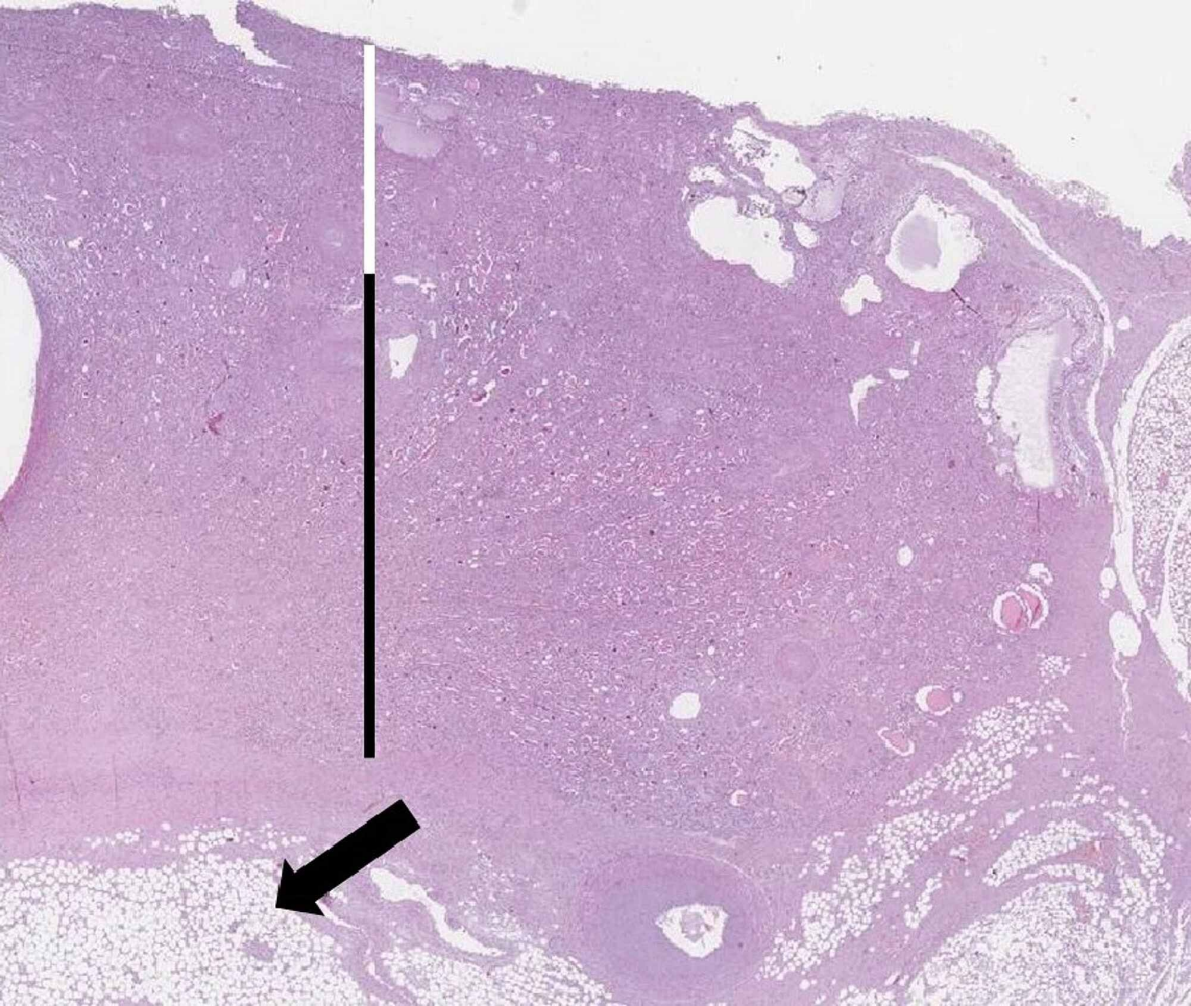
Histotopographic section of the kidney Cortex (white line), medulla (black line), and renal pelvis adipose tissue (arrow) are seen (hematoxylin and eosin stain).

**Figure 4:**
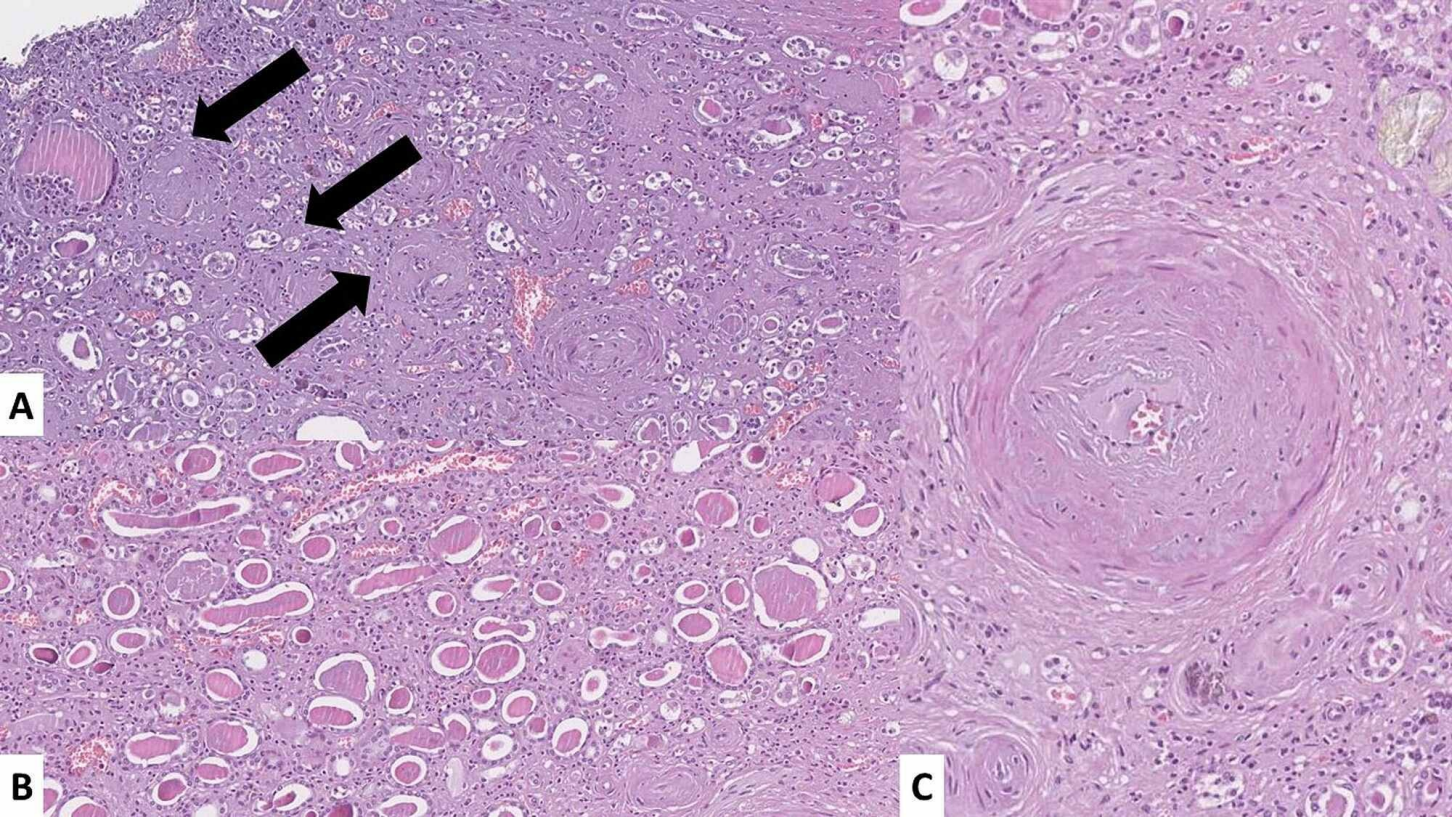
Kidney histopathology (A) Sclerotic glomeruli (arrows) in the superficial part of the cortex with diffuse interstitial sclerosis (H&E stain, original magnification 100x); (B) areas of thyroidization, diffuse interstitial sclerosis, and scant inflammatory cells (H&E stain, original magnification 100x); (C) arteriosclerosis, interstitial sclerosis, and scattered inflammatory cells (H&E stain, original magnification 100x) H&E, hematoxylin and eosin

Renal histology revealed a decreased cortical thickness, diffuse glomerular sclerosis in the superficial part of the cortex, thyroidization of the renal medulla, severe vascular changes with hyalinization, arteriosclerosis, and vascular obliteration, with diffuse interstitial sclerosis and scattered lymphocytic inflammatory cell infiltration (Figures 3, 4).

**Figure 5:**
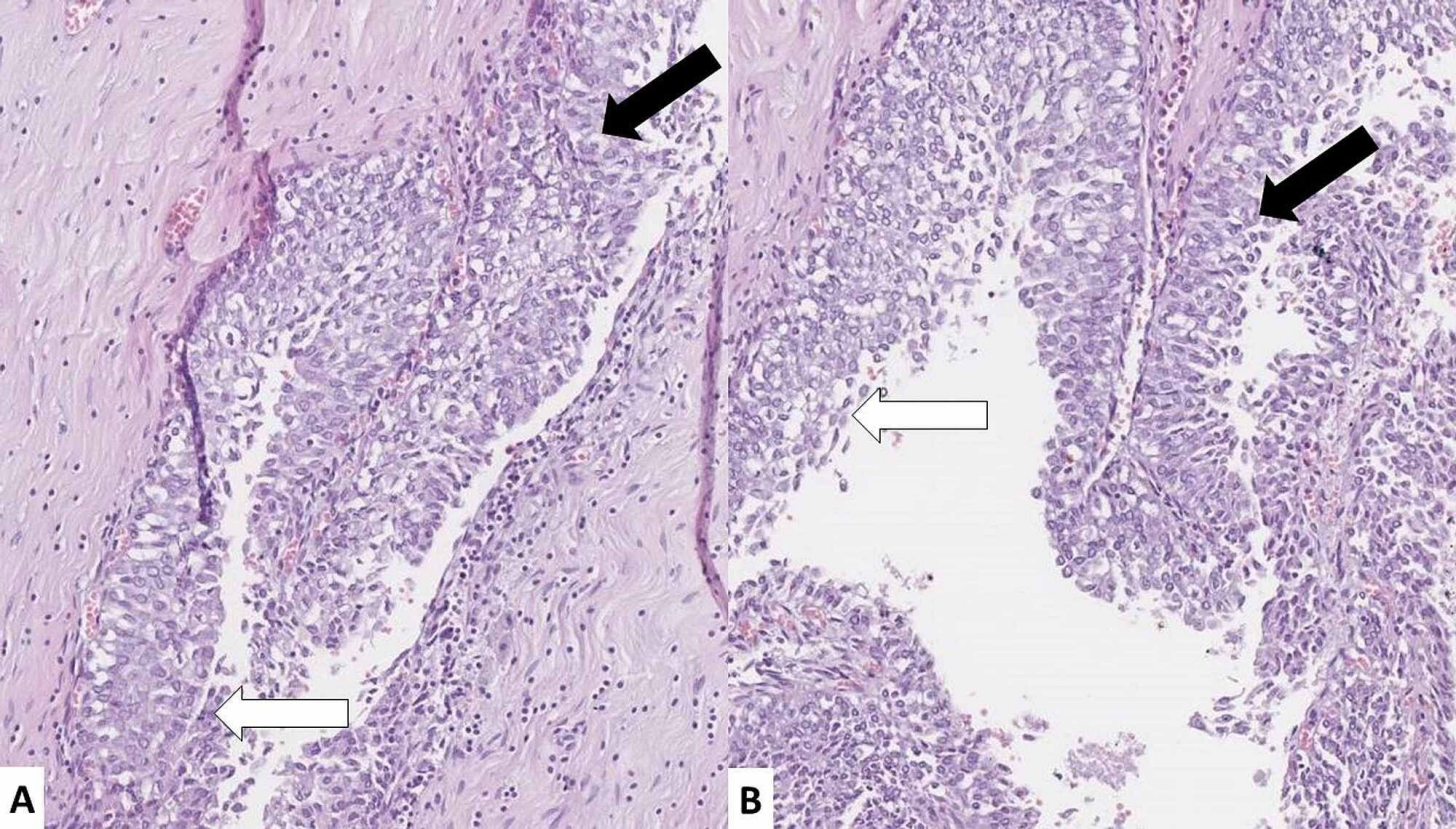
Ureter histopathology (A and B) Urothelial papilloma’s (black arrows) and atypical urothelial hyperplasia (white arrows); hematoxylin and eosin stain, original magnification 100x

The ureters histologically revealed not only multiple papillomas but also diffuse atypical urothelial hyperplasia (Figure 5).

The rest of the gross and histological findings were uneventful other than scattered areas of fibrinous inflammation of the serous membranes due to the severe uremia.

## Discussion

Despite local descriptions dating back to as early as 1953, the first official report of 665 patients was published in 1956 [1]. The condition was originally referred to as Vratza nephropathy, as cases were only observed in small villages in the area. Several years afterward, similar patients were described in the modern-day Serbia and Romania, which referred to the condition as Tanchev’s nephropathy [1-3]. With time, however, more endemic areas around the Danube were described; however, all of them were located on the Balkan peninsula, and hence the condition became known as BEN [3].

The etiology of the condition had been widely disputed with genetic, toxic, and infectious agents being the most widely discussed. Recent data has suggested that BEN develops due to long-lasting exposure to aristolochic acid, due to the virtually identical clinical and morphological manifestation of aristolochic acid nephropathy (AAN), also referred to as Chinese herb nephropathy (CHN) [4-8]. It was not until the 1990s that patients in Belgium started developing interstitial nephritis after taking food supplements consisting of Chinese herbs containing aristolochic acid, and later urothelial malignancies [4-6]. Later, when the clinical and histological changes in BEN and AAN/CHN were evaluated, it was established that they were identical [6]. Indeed, nowadays these conditions are viewed as the same nosological unit, developing in different geographical areas, due to dietary supplementation, traditional herbal remedies, or environmental contamination of drinking water.

Several other forms of endemic nephropathies have also been described recently, with some harboring clinical similarities to BEN-CHN-AAN, such as ochratoxin nephropathy in Tunisia, chronic kidney disease of unknown etiology in Sri Lanka, and Mesoamerican nephropathy, which may present as other geographical variants of the same disease (Table 1) [4-7,9-14].

**Table 1:** Comparison of the features of different forms of geographical variants of interstitial nephritis BEN, Balkan endemic nephropathy; AAN, aristolochic acid nephropathy; CHN, Chinese herb nephropathy; ON, ochratoxin nephropathy; CKDUE, chronic kidney disease of unknown etiology; MN, Mesoamerican nephropathy

Condition	Geographical area	Features	Etiology	Reference
BEN	Balkans	Glomerulosclerosis in the external part of the cortex, tubular atrophy, and interstitial fibrosis with scant inflammation, high incidence of urothelial neoplasia	Aristolochic acid, patients are from endemic rural areas, aged 20-60 years	[1,2,4,11]
AAN/CHN	Initially Belgium, now international	Identical to BEN	Aristolochic acid, predominantly young females	[4-7,11]
ON	Tunisia	Identical to BEN, no reported urothelial neoplasia	Ochratoxin A, patients in their fourth and fifth decade	[11,12]
CKDUE	Sri Lanka	Tubular atrophy, interstitial fibrosis and inflammation in the cortex, glomerular hyalinization	Unknown, most patients are males aged 20-69 years, agricultural workers (irrigation water contaminated with heavy metals and chemicals has been suspected)	[10,11,13]
MN	Central America	Tubular atrophy, interstitial fibrosis, glomerular hyalinization, hypertrophic glomerular tuffs	Unknown, most patients are young males working in the agriculture sector (excluding coffee plantations)	[9-11,14]

The increasing unregulated use of food supplements can in the future lead to an increased incidence of these conditions in non-endemic areas and can have severe life-threatening complications for the patients’ lives not only due to the development of chronic renal failure but also to the development of urothelial malignancies.

The depicted changes in our case underline the gross and histomorphological changes associated with BEN-CHN-AAN, with the classical complex of renal pathology.

## Conclusions

BEN is a form of interstitial nephritis associated with urothelial malignancies. The condition is likely due to the longstanding exposure to aristolochic acid and is a variant of BEN-CHN-AAN. The described gross and histological changes are specific for this complex condition.
